# Evaluation of Guava Pulp Microencapsulated in Mucilage of Aloe Vera and *Opuntia ficus-indica* as a Natural Dye for Yogurt: Functional Characterization and Color Stability

**DOI:** 10.3390/foods11152380

**Published:** 2022-08-08

**Authors:** Maria Carolina Otálora, Andrea Wilches-Torres, Jovanny A. Gómez Castaño

**Affiliations:** 1 Grupo de Investigación en Ciencias Básicas (NÚCLEO), Facultad de Ciencias e Ingeniería, Universidad de Boyacá, Tunja 050030, Boyacá, Colombia; 2 Grupo Química-Física Molecular y Modelamiento Computacional (QUIMOL®), Escuela de Ciencias Químicas, Universidad Pedagógica y Tecnológica de Colombia, Sede Tunja, Avenida Central del Norte, Tunja 050030, Boyacá, Colombia

**Keywords:** microencapsulation, spray drying, yogurt, natural colorant, aloe vera, *Opuntia ficus-indica*

## Abstract

The substitution of artificial colorants for pigments extracted from fruits is a highly desirable strategy in the food industry for the manufacture of natural, functional, and safe products. In this work, a 100% natural spray-dried (SD) microencapsulated colorant of pink guava pulp, using aloe vera (AV) or *Opuntia ficus-indica* (OFI) mucilage as functional encapsulating material, was prepared and evaluated as an additive into a yogurt (Y) matrix. The characterization of yogurt samples supplemented with OFI (Y-SD-OFI) and AV (Y-SD-AV) mucilage-covered guava pulp microcapsules was carried out through carotenoid quantification using UV–vis and HPLC–MS techniques, dietary fiber content, antioxidant capacity, colorimetry, and textural analysis, as well as by an evaluation of color stability after 25 days of storage at 4 °C in the dark. These physicochemical characteristics and color stability on the Y-SD-OFI and Y-SD-AV samples were compared with those of a commercial yogurt (control sample, Y-C) containing sunset yellow FCF synthetic colorant (E110). Y-SD-OFI and Y-SD-AV samples exhibited a high content of lycopene, dietary fiber, and antioxidant activity, which were absent in the control sample. Microencapsulated lycopene imparted a highly stable color to yogurt, contrary to the effect provided by the E110 dye in the control sample. The texture profile analysis revealed an increase in firmness, consistency, and cohesion in the Y-SD-OFI sample, contrary to the Y-SD-AV and Y-C samples, which was attributed to the variation in fiber concentration in the microcapsules. The incorporation of OFI and AV mucilage microparticles containing pink guava pulp into yogurt demonstrated its potential application as a functional natural colorant for dairy products.

## 1. Introduction

Color is an essential attribute of a food product intended to provide sensory quality to the consumer translated in terms of freshness, safety, stimulation, and acceptability. For decades, the world food industry has been inclined toward the use of synthetic dyes as additives to give color and a pleasant appearance to foods, due to their high chemical and physical stabilities, coloring power, and, mainly, low costs [1]. However, synthetic additives are becoming less acceptable to consumers due to their possible associated harmful effects, including allergies, irritability, attention deficit, genotoxicity, and carcinogenicity [2]. For instance, sunset yellow FCF (E110) is an orange water-soluble anionic monoazo dye, widely applied in the food industry, the use of which is questioned due to its associated side effects, among which are hyperactivity in childhood, atopic dermatitis, urticaria, and human angioedema [2]. In response to these limitations, modern consumers have a growing food tendency to choose products with natural colorants that promote beneficial effects on their health.

Nature offers us a wide variety of products that can be used as functional food colorants. Among these, we find carotenoids and lipophilic pigments responsible for the yellow, orange, and red colors of plant foods (present in leafy vegetables, non-leafy vegetables, and fruits) and for the red and yellow colors in some animal tissues (e.g., fish meat, crustaceans, and egg yolk). β-carotene, α-carotene, β-cryptoxanthin, lutein, zeaxanthin, and lycopene are the carotenoids most found in natural products [3]. Beneficial effects on human health are attributed to these types of natural compounds due to their high antioxidant, photoprotective, and provitamin A activities [4]. Among the main causes that prevent the massive use of carotenoids from natural products in the food industry is their low resistance to factors such as changes in pH, high temperatures, oxygen, and exposure to light [4,5]. These factors lead to reduced shelf life and the low bioavailability of these natural colorants when added directly to a food matrix.

Alternatively, spray drying (SD) has been the most widely used microencapsulation method in the pharmaceutical, cosmetic, and food sectors to trap carotenoids, mainly due to its ease in producing solids from liquids, converting hydrophobic compounds into water-dispersible powders, and improving the stability of bioactive compounds sensitive to extrinsic factors, thus increasing their useful life [4,6]. In this context, we have recently microencapsulated the pulp of pink guava (a rich source of lycopene with high antioxidant capacity) using mucilage from the cladodes of *Opuntia ficus-indica* and aloe vera leaves as encapsulating material [7]. The selection of these biopolymers as wall materials in the microcapsules was made due to their antioxidant functional properties and high content of dietary fiber [8,9], which was used to replace maltodextrin, a conventional encapsulating material based on polysaccharides that does not provide a nutritional value added to the final product [10]. The findings of our study revealed that red guava pulp microcapsules can be promising sources of biofunctional natural ingredients (colorant with antiradical power) with the ability to be incorporated into food matrices that lead to the generation of new food products with high nutritional value, natural color, and antioxidant capacity [11].

In the last decade, there has been considerable interest in fortifying milk and dairy products with a wide variety of functional ingredients [12]. However, only a few studies related to the incorporation of microencapsulated carotenes in real dairy foods have been reported, due to the incidence of encapsulating agents and the concentration of the microencapsulated compounds in color and potential contributions to human health, as well as undesirable modifications, including changes in the texture, rheology, and flavor of the product [4,13]. Among the few studies on the successful application of carotenoid-rich microparticles in dairy products are the works by Rutz et al. [14] and Gómez-Estaca et al. [15] for yogurt and Mihalcea et al. [16] for ice cream.

Yogurt is one of the most popular dairy products because of its unique organoleptic and nutritional properties. However, this fermented matrix is not considered a rich source of bioactive compounds, which makes it interesting as a food matrix for the addition of red guava pulp microcapsules rich in lycopene. In this study, the objective was to evaluate the incorporation of microencapsulated carotenoids in natural yogurt and measure the effects on carotenoid quantification (UV–vis and HPLC–MS), and colorimetric and textural quality, as well as the antioxidant properties and total dietary fiber content, resulting from this addition. For comparative analysis, the studies carried out on yogurt enriched with microcapsules were also conducted on a control sample of commercial yogurt containing a synthetic dye.

## 2. Materials and Methods

### 2.1. Vegetal Materials

Guava fresh fruits (*Psidium guajava* L.) of the Palmira ICA-1 regional variety, cladodes of *Opuntia ficus-indica*, and aloe vera leaves were purchased at a local supermarket in the city of Tunja in the Department of Boyacá, Colombia. Similarly, two commercial samples of the same brand of natural yogurt were purchased in the local market of the city of Tunja in the Department of Boyacá, Colombia, one without the addition of artificial colorant and the other containing sunset yellow (E110) as a synthetic dye.

### 2.2. Extraction of Pulp Guava and Mucilages

The preparation of pulp guava was carried out following the methodology reported by Otálora et al. [7]. The fruit units were washed with distilled water and manually peeled to obtain the pulp and seeds. The pulp was immediately chopped and homogenized in a blender at minimum power for 1 min. The pulp was sieved to remove the seeds; then, it was frozen at −80 °C in an ultra-low temperature freezer (Buzzer, model MDF–86V188E, Shanghai, China) for 48 h. Afterward, the samples were freeze-dried in a Freezone 4.5 L freeze dryer (Labconco, Kansas City, MO, USA) at −84 °C in a vacuum of 0.13 mbar for 48 h. After freeze drying, the samples were triturated using a food processor and stored in amber bottles until further use.

**Figure 1:**
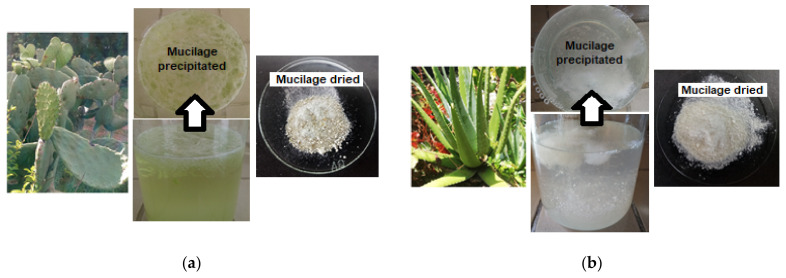
Appearance of the purified mucilage: (**a**) mucilage extracted from OFI cladodes; (**b**) mucilage extracted from Aloe Vera leaves.

To extract mucilage from the cladodes of *Opuntia ficus-indica* and aloe vera leaves, the methodology reported by Quinzio et al. [17] and Otálora et al. [18] was used, with some modifications. Slices of the medulla, obtained by removing the epidermis from cladodes and aloe vera leaves, were cut into small pieces and then placed separately into two beakers. Distilled water at room temperature was added to each beaker until reaching a ratio of 1:2 *v*/*v* (medulla:water). The mixtures were left for 12 h and then manually squeezed to extract the gel, which was immediately filtered. Then, 95% ethanol was added to each filtered gel until it reached a ratio of 3:1 (ethanol:filtered gel) at room temperature with constant agitation. The precipitated mucilages (white-milky gels) were placed in Petri dishes and dried in an oven (UM 400, Memmert, Schwalbach, Germany) for 12 h at 50 °C (cladodes mucilage) and 105 °C (aloe vera leaves mucilage). The dried materials were manually macerated in a porcelain mortar until a fine powder was obtained. Photographs of mucilage extracted from OFI cladodes and AV leaves are presented in Figure 1.

### 2.3. Spray-Drying Microencapsulation of Guava Pulp in AV and OFI Mucilages

**Figure 2:**
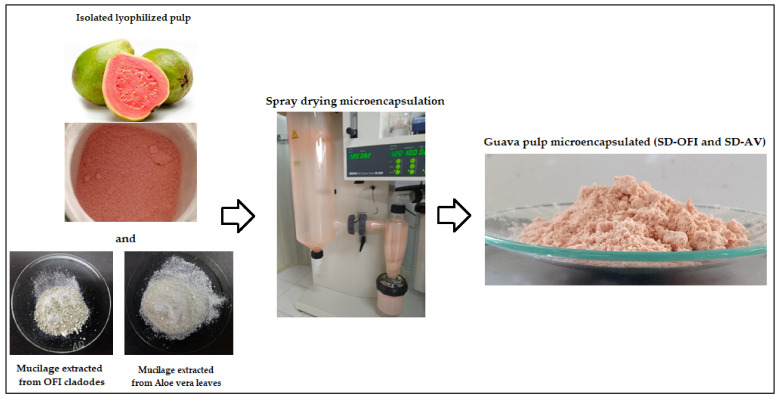
Spray-drying microencapsulation process of Guava Pulp in AV and OFI mucilages.

The microencapsulation of lyophilized guava pulp via spray drying was performed according to the methodology reported by Otálora et al. [7]. Aqueous solutions of mucilage from cladodes of *Opuntia ficus-indica* (1.2 g/100 mL) and aloe vera leaves (0.4 g/100 mL), as wall materials, were prepared at 18 °C by constantly stirring at 300 rpm for 2 h using a magnetic stirrer (C-MAG HS 7 S000, IKA, Staufen im Breisgau, Germany). Then, 10 g of lyophilized guava pulp was added to each mucilage solution (100 mL), and then each mixture was kept under constant magnetic stirring at room temperature until homogeneity. The total solids content of the feed mixes was 5.55% for SD-AV (guava pulp/AV mucilage) and 4.34% for SD-OFI (guava pulp/OFI mucilage) mixtures. The feed mixture was subjected to a mini spray dryer (B-290, Büchi Labortechnik, Flawil, Switzerland) with an aspiration maintained at 100% (35 m^3^/h) to maximize the separation rate of the cyclone [19] and a compressed air pressure of 40 bar, using an internal diameter of 0.7 mm, a feed flow of 350 mL/h, and an inlet air temperature of 120 °C. The two microencapsulated samples that were obtained from this procedure, i.e., SD-OFI and SD-AV, were stored in the dark at −20 °C for subsequent analysis and use. Photographs of the spray-drying microencapsulation process of guava pulp in AV and OFI mucilages are presented in Figure 2. 

### 2.4. Addition of Guava Pulp Microcapsules in Yogurt

To test the microencapsulated guava pulp powders as a functional colorant, 3.0 g of SD-OFI and 2.0 g of SD-AV were separately added to two samples of commercial yogurt (compound of pasteurized skim milk, *Lactobacillus delbrueckii* subsp. *bulgaricus* and *Strep. thermophilus* and without artificial dyes) of 100 mL each. These quantities of guava pulp powder were established based on the color change necessary to achieve a similar appearance in the test yogurt samples to that of the control sample. Both yogurt samples added with microcapsules (Y-SD-OFI and Y-SD-AV) were homogenized with constant stirring at 300 rpm for 30 min using a magnetic stirrer (C-MAG HS 7 S000, IKA, Staufen im Breisgau, Germany). Y-SD-OFI and Y-SD-AV samples were placed in amber glass vials and stored at 4 °C until characterization.

The nutritional information of the commercial yogurt, before the addition of the microencapsulated natural dye, reports the following values: energy = 292 kJ (0.07 kcal); lipids = 1.5 g, saturated = 1.0 g; carbohydrates = 9.0 g; sugars = 0.0 g; protein = 5 g; and dietary fiber = 0.0 g. As a control sample, commercial yogurt added with sunset yellow (E110) synthetic colorant was considered, which presented the same nutritional information values as the yogurt sample without the synthetic colorant.

### 2.5. Yogurt Analyses

#### 2.5.1. Total Carotenoid Content

Total carotenoid content determination was performed according to the methodology reported by Otálora et al. [7]. Carotenoids were extracted with 10 mL acetone by separately macerating 208 mg of Y-SD-OFI, 212 mg of Y-SD-AV, and 354 mg of Y-C samples and then stirred for 1 min at room temperature and filtered through a Millipore membrane (0.45 µm). Carotenoids were quantified by a UV–vis spectrophotometer (V530, Jasco, Hachioji, Tokyo, Japan) at 450 nm wavelength. The results in each yogurt sample are expressed in micrograms of β-carotene per gram of sample.

#### 2.5.2. Lycopene and β-Carotene Quantification by HPLC–MS

The quantification of Lycopene and β-Carotene was performed according to the methodology reported by Otálora et al. [7]. The Y-SD-OFI, Y-SD-AV, and control yogurt samples were dissolved in dichloromethane and n-hexane (1:1, *v*/*v*), filtered through a 0.45 μm pore size nylon Millipore and injected into the Acquity ultraperformance liquid chromatography (UPLC) system equipped with a Xevo TQ-XS Mass Spectrometer (Waters Corp., Milford, MA, USA) and an electrospray ionization (ESI) probe that was operated in the positive ion mode to detect individual carotenoids (lycopene and β-carotene). An ACQUITY UPLC BEH C18 analytical column (2.1 mm × 100 mm, 1.7 µm particle size) was used for the analysis of the analytes present in each sample, using a column temperature of 40 °C and a flow rate of 0.4 mL/min. The solvent mixture consisted of acetonitrile (solvent A) and methanol (solvent B). The gradient elution program was set as follows: 0–5 min, 100% A; 5.0–8.5 min, 80% B and 20% A; and 8.5–12 min, 100% A. The injection volume was set at 2 µL. MS parameters were as follows: the capillary voltage was set at 2.5 kV, while blocking and desolvation temperatures were set at 150 °C and 400 °C, respectively. The desolvation gas flow rate was set at 800 L/h, and the cone gas was set at 50 L/h. Cone voltages were set to 68 and 64 V, and collision energies were set to 60 and 52 eV for lycopene and β-carotene, respectively.

#### 2.5.3. Color Parameters and Color Storage Stability

Yogurt samples containing microencapsulated carotenoids (i.e., Y-SD-OFI and Y-SD-AV) and synthetic colorant (Y-C) were stored for 25 days at 4 °C in the dark. For each sample, the color attributes (L*, a*, and b*) and color parameters chroma ($C_{ab}^{*}$) (Equation (1)) and hue ($h_{ab}$) (Equation (2)) were measured at the initial time (zero time) and after 25 days of storage using a Chroma Meter CR-300 (Konica Minolta Co., Osaka, Japan).

(1) $$C_{ab}^{*}={\left[{\left(a^{*}\right)}^{2}+{\left(b^{*}\right)}^{2}\right]}^{1/2},$$

(2) $$h_{ab}=arctan\;\left[b^{*}/\;a^{*}\right],$$

The total color change ($\Delta E^{*}$) in each sample was calculated using Equation (3).

(3) $$\Delta E^{*}=\sqrt{{\left(L_{0}^{*}-L_{25}^{*}\right)}^{2}+{\left(a_{0}^{*}-a_{25}^{*}\right)}^{2\;\;\;}+{\left(b_{0}^{*}-\;b_{25}^{*}\right)}^{2}},$$

where $L_{0}^{*}$, $a_{0}^{*}$, and $b_{0}^{*}$ are the color parameters at zero time, and $L_{25}^{*}$, $a_{25}^{*}$, and $b_{25}^{*}$ are the respective values after 25 days of storage.

### 2.6. Functional Properties

#### 2.6.1. Antioxidant Capacity

The Trolox equivalent antioxidant capacity (TEAC) was measured using the method reported by Re et al. [20]. Around 210 mg of Y-SD-OFI, 208 mg of Y-SD-AV, and 210 mg of Y-C yogurt samples were dissolved in 10 mL of methanol, stirred at room temperature, and filtered through a Millipore membrane. The 0.5 µL liquid samples were recovered and mixed with 1 mL ABTS^•^ solution, and their absorbance was read at 734 nm, measured using a UV–vis spectrophotometer (V530, Jasco, Hachioji, Tokyo, Japan) with methanol as blank. The results are expressed in millimoles of Trolox equivalents per kilogram of sample (TEAC).

#### 2.6.2. Dietary Fiber Content

The total dietary fiber content (TDFC) in Y-SD-OFI, Y-SD-AV, and Y-C yogurt model samples was determined using a total dietary fiber test kit (TDF-100A), provided by Sigma-Aldrich (St. Louis, MO, USA), which is based on the enzymatic–gravimetric method AOAC 985.29 [21].

#### 2.6.3. Textural Properties

The texture profiles (firmness, consistency, and cohesiveness) of Y-SD-OFI, Y-SD-AV, and Y-C samples were determined using a TA-XT plus Texture Analyzer (Stable Micro Systems, Surrey, UK) and the accompanying software (Exponent). Yogurt samples at 4 ± 1 °C were compressed under a cylindrical probe (AB/E-35, diameter 35 mm) at a test speed of 1 mm/s, a penetration depth of 30 mm, and a trigger force of 1.0 g. In these analyses, firmness is related to the force required to achieve the maximum depth, while cohesiveness refers to the force required to pull the probe away from the sample.

### 2.7. Statistical Analysis

Carotenoid content, antioxidant properties, and color parameter data, as presented in Table 1 and Table 2, are reported as means ± standard deviations (*n* = 3). The Kruskal–Wallis’s test was performed to identify differences among the means using the InfoStat/P version 1.1 statistical software [22]. Differences at probability level *p* < 0.05 were considered significant.

## 3. Results and Discussion

### 3.1. Total Content of Carotenoids and Fiber, and Antioxidant Capacity

As shown in Table 1, the addition of guava pulp microencapsulated in mucilage from OFI cladodes and AV leaves to yogurt samples (Y-SD-OFI and Y-SD-AV) led to an increase in their total carotenoid content (TCC) by about 7.2 and 6.4 times, respectively, compared with the yogurt sample with synthetic E110 dye (Y-C). As the baseline of 10.6 µg β-carotene/g in the yogurt sample without guava pulp microcapsules (Y-C) is associated with the intrinsic content of β-carotene in the milk used to make the yogurt product [23], the excess of TCC in the samples Y-SD-OFI and Y-SD-AV must correspond to the presence of lycopene from the guava pulp.

The increase in TCC in the Y-SD-OFI and Y-SD-AV yogurt samples contributed to an almost directly proportional response in their antioxidant capacity (measured as TEAC) at a rate of 6.6 and 5.5 times with respect to the control sample (Y-C). Similar results were found in other studies in which the yogurt matrix was supplemented with lipophilic antioxidants, such as carotenoids [24,25]. As we recently reported [7], the TEAC activity provided by guava pulp/OFI mucilage and guava pulp/AV mucilage microcapsules is a joint response between guava carotenoid content and mucilage polyphenol content. The higher TCC and TEAC values of the Y-SD-OFI sample, compared with the Y-SD-AV sample, were mainly attributed to the higher microcapsule dye content with OFI mucilage added to the yogurt matrix (3.0 g/100 mL in Y-SD-OFI vs. 2.0 g/100 mL in Y-SD-AV).

The enrichment of the fiber content (TDFC) was also revealed in the yogurt samples added with microcapsules of guava pulp (Table 1), while the yogurt sample containing dye E110 had zero dietary fiber content. The origin of the dietary fiber content in the Y-SD-OFI and Y-SD-AV samples was directly associated with the fiber content provided by the mucilage biopolymers that make up the microcapsule walls [18]. The reported value of dietary fiber in the isolated microcapsules of guava pulp covered with OFI and AV mucilages was 32.1 ± 0.1 g/100 g and 22.8 ± 0.1 g/100 g [7], respectively, which explains the higher value of TDFC in the Y-SD-OFI sample (12.8 g/100 g) than that of the Y-SD-AV sample (8.3 g/100 g).

### 3.2. Quantification of Carotenoid Content by HPLC–MS

**Figure 3:**
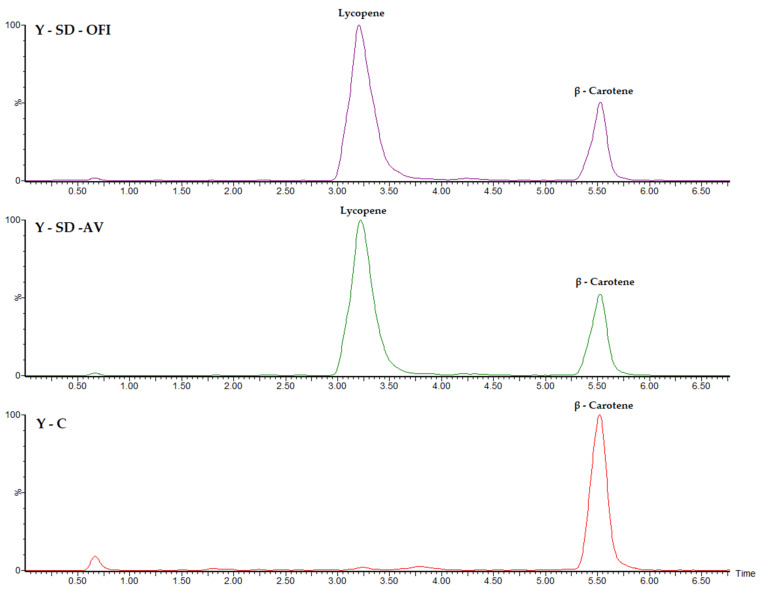
HPLC–MS chromatograms of the Y-SD-OFI, Y-SD-AV, and Y-C yogurt samples.

The HPLC–MS technique allowed us to specify the identity of the carotenoids present in the Y-SD-OFI, Y-SD-AV, and Y-C yogurt samples. As revealed in the chromatograms presented in Figure 3, the yogurt sample added with the artificial dye E110 showed a signal at a retention time of 5.5 min, corresponding to the presence of β-carotene at a concentration of 128 mg/kg dry sample (Table 1). This concentration of β-carotene was very close to that detected in Y-SD-OFI (127 mg/kg dry sample) and Y-SD-AV (124 mg/kg dry sample) samples; therefore, its content must have been provided by the milk used as raw material. The content of β-carotene in milk is the result of the presence of this natural pigment in plants consumed by cattle [23,26]. β-carotene is generally found dispersed in milk, and part of it is naturally bound to milk proteins, which is why the breaking of this ligand-protein bond by physicochemical factors is necessary to achieve accurate β-carotene quantification in milk samples [23,26].

The HPLC chromatograms of the Y-SD-OFI and Y-SD-AV yogurt samples (Figure 3) showed that the extra content of carotenoids in these dairy matrices was due to the presence of lycopene provided by the guava pulp. The mass quantification of lycopene signals at a retention time of 3.2 min led to the determination of concentrations of 352 mg/kg dry sample for Y-SD-OFI and 346 mg/kg dry sample for Y-SD-AV (Table 1). Given that no signals attributable to lycopene decomposition compounds were detected in the chromatograms of the Y-SD-OFI and Y-SD-AV yogurt samples, a high protective effect of both mucilage envelopes in the guava pulp microcapsules added to these dairy matrices was demonstrated.

### 3.3. CIELab Color Space

The color parameters listed in Table 2 showed that the luminosity value (L*) did not present a significant difference between the yogurt samples supplemented with pulp microcapsules (57.7 for Y-SD-OFI vs. 56.1 for Y-SD-AV), which was attributed to the white color that characterizes both mucilage biopolymers that make up the microcapsule wall (Figure 1). However, the yogurt sample added with the synthetic colorant had a slightly higher L* value (about 4 and 6 units above, respectively) than the Y-SD-OFI and Y-SD-AV samples because of the high luminosity provided by dye E110 to the products in which it is added (e.g., 68.02 ≤ L* ≤ 62.76) [27].

The Y-SD-OFI and Y-SD-AV samples showed close values between their color parameters a* and b* (Table 2), although with opposite behavior. The Y-SD-AV sample had a slightly more reddish value (a* = 3.15) than the Y-SD-OFI sample (a* = 2.84), while the latter had a slightly more yellowish value (b* = 11.91) than the former one (b* = 9.79). This color behavior agreed with the slightly higher presence of β-carotene and lower lycopene content in the Y-SD-AD sample than in the Y-SD-OFI sample (Table 1). Both a* and b* values in the samples enriched with guava pulp microcapsules presented significant differences with respect to the values for these coordinates in the sample with artificial dye. Sample Y-C exhibited an a* value of 13.74, corresponding to a higher reddish value, and a high value in its yellow coordinate (b* = 77.72), coinciding with what was expected for the sunset yellow dye (E110).

The chroma parameter ($C_{ab}^{*}$) was significantly lower (*p* < 0.05) for the yogurt samples supplemented with guava pulp/mucilage microcapsules (12.24 for Y-SD-OFI and 10.29 for Y-SD-AV) than for the yogurt sample added with the pigment E110 (78.92). Chroma parameter ($C_{ab}^{*}$) expresses the degree of the color of a viewed area relative to its brightness and is greater when the color coordinates are farther from the CIEL*ab* coordinate origin. Consequently, the lower values of $C_{ab}^{*}$ in the Y-SD-OFI and Y-SD-AV samples, compared with that of the Y-C sample, were associated with the lower saturation and color intensity provided by the lycopene pigment present in yogurt samples enriched with pink-guava microcapsules.

**Figure 4:**
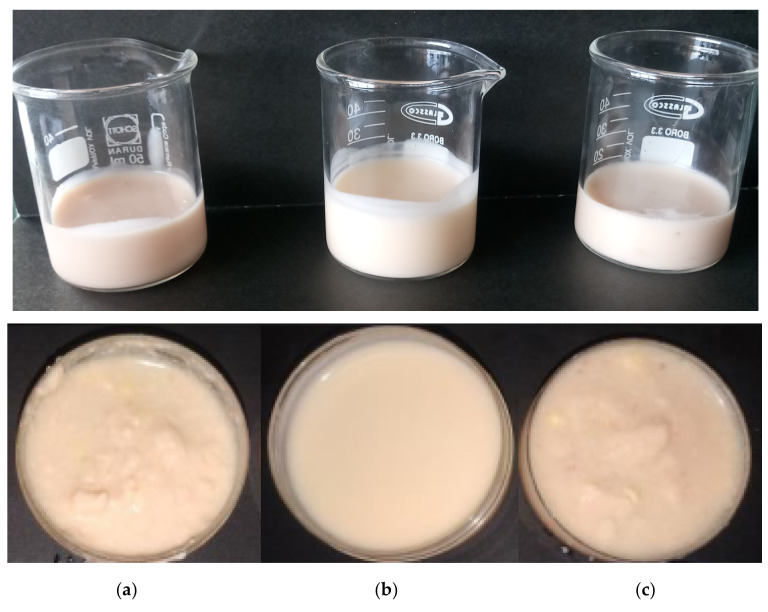
Appearance comparison among Y-SD-OFI (**a**), Y-C (**b**), and Y-SD-AV (**c**) before (**top** row) and after storage for 25 days in the dark at 4 °C (**bottom** row).

Although the samples added with guava/mucilage microcapsules presented values of a* and b* far from those of the sample with pigment E110, the value of the tonality parameter ($h_{ab}$) presented close values among these three samples (Table 1), which indicated a common yellowish hue in the three types of yogurt matrices (Figure 4).

### 3.4. Storage Stability Analysis

The last row of Table 2 contains the magnitude of the total color difference achieved in the Y-SD-OFI, Y-SD-AV, and Y-C yogurt samples after 25 days of storage in the dark at 4 °C. Yogurt samples supplemented with guava pulp microcapsules showed significantly less total color change, about eight (Y-SD-OFI) and five (Y-SD-AV) times lower than that of the sample added with the artificial dye (E110). The coordinate that contributed the most to the total color change in yogurt samples supplemented was b* (yellow coloration) from 11.80 ± 0.12 to 15.82 ± 0.35 and 9.79 ± 0.55 to 13.94 ± 0.65 for Y-SD-OFI and Y-SD-AV at 0 and 25 days of storage, respectively, as result of the protective effect of the polymer (mucilage) in the pigment during storage time of yogurt [28]. However, sample Y-C presented a substantial change in its total color after storage (∆*E* = 63.32 ± 2.64) as a result of a significant variation in the three color coordinates L* (∆L* = 7.43), a* (∆a* = 9.96), and b* (∆b* = 62.08), which caused opacity and loss of the yellow hue in this sample during storage time. This was associated with the chemical instability of artificial colorant E110 in the milk matrix [29,30]. Similar results were reported by Bassijeh et al. [31], who found that samples of a model drink added with dye E110 presented a considerable deterioration in their color after two months of storage in the dark at 4 °C, while the samples of the same drink added with astaxanthin microcapsules retained their color properties under these storage conditions.

Therefore, the use of lycopene as a coloring ingredient increased the intensity of the yellow hue in these food matrices during storage. This behavior is likely because carotenoids undergo changes in their chemical structure that lead to the formation of new chemical interactions (hydrophobic bonds, van der Waals interactions, and hydrogen bonds), which, in turn, lead to the greater availability of atoms of hydrogen, both for the transport of electrons and to promote the neutralization of reactive oxygen species, increasing the antioxidant activity and, therefore, the content of carotenoids [32]. Similar behavior was reported by Chung et al. [33], who showed that the use of denatured whey protein can prolong the anthocyanin color stability in model beverages. Likewise, Estupinan et al. [34] demonstrated that the addition of maltodextrin as a carrier agent stabilized the color of isotonic model beverages developed with lyophilized anthocyanin.

As can be seen in the lower photographs of Figure 4, the Y-SD-OFI and Y-SD-AV samples showed an increase in their coarseness (i.e., lumps) after 25 days of storage at 4 °C. Such behavior is the result of the formation of mucilage biopolymer networks by hydrogen bonding with water molecules in the medium (gelation) [35,36]. Although this phenomenon leads to a different sensory experience for the consumer than the one traditionally offered by a sample added with a synthetic colorant (e.g., Y-C), such particularity can be directed toward the formation of Greek-type yogurt products.

### 3.5. Firmness, Consistency, and Cohesiveness

As shown in Table 3, the incorporation of OFI-mucilage-coated guava pulp microcapsules increased the texture profile of the yogurt sample compared with the Y-SD-AV and Y-C samples. This behavior was attributed to the higher daily fiber content present in the Y-SD-OFI sample (Table 1), which favored the formation of three-dimensional structures by an interaction between fiber and casein aggregates. Such aggregates promote greater resistance to deformation, i.e., they strengthen the gel structure, thus preventing the separation of serum and, therefore, a reduction in syneresis [37,38].

Alternatively, the Y-SD-AV yogurt sample presented lower firmness, consistency, and cohesion than the sample added with the synthetic dye (E110). The incorporation of AV-mucilage-coated guava pulp microcapsules probably caused the rearrangement of the gel structure within the yogurt sample, which was accompanied by a loss of water retention capacity (contraction) and whey expulsion from the yogurt [35]. Similar behavior was reported by C. de Campo et al. [11], who showed that the addition of zeaxanthin nanoparticles in yogurt samples promoted a decrease in their firmness and consistency.

Overall, the texture profile of yogurts supplemented with guava mucilage/pulp microcapsules, i.e., Y-SD-OFI (1.2 g mucilage) and Y-SD-AV (0.4 g mucilage), can be correlated with its mucilage concentration. The mucilage content in these samples increased their texture profile while decreasing their syneresis [36]. Therefore, the particle diameter of 26.9 μm [6] and the number of particles added to the Y-SD-OFI yogurt were adequate to achieve the desired sensory attributes of the product (i.e., color and texture) [4].

## 4. Conclusions

In this study, two varieties of a new microencapsulated natural dye, using mucilage from *Opuntia ficus indica* (OFI) cladodes (variation 1) or from aloe vera (AV) leaves (variation 2) as wall material, were used as both carrier and protector media for the incorporation of pink guava pulp into yogurt matrices. The measurement of the total content of carotenoids (TCC) and dietary fiber (TDFC), antioxidant capacity (TEAC), CIELab color space analysis, and the quantification of lycopene and β-carotene via HPLC–MS, as well as the study of the texture profile (firmness, consistency, and cohesion), allowed us to determine the functional and some physicochemical properties of yogurt samples enriched with microcapsules of guava pulp/OFI mucilage (sample Y-SD-OFI) or guava pulp/AV mucilage (sample Y-SD-AV). Likewise, the colorimetric stability of the Y-SD-OFI and Y-SD-AV samples was evaluated after 25 days of storage in the dark at 4 °C. As a control sample, commercial yogurt added with the artificial color sunset yellow (E110) was used for all studies.

Our results showed that the incorporation of microencapsulated guava pulp in OFI or AV mucilage provided the yogurt samples with high lycopene content, greater antioxidant capacity, and incorporation of dietary fiber, contrary to what was observed in the sample added with the artificial dye (E110). HPLC–MS measurements, along with the colorimetric stability study, showed the efficient protective capacity of the guava pulp content provided by the mucilage-encapsulating material. As a result, the color characteristics in the yogurt samples supplemented with microcapsules were maintained during the storage period, while the sample added with dye E110 lost its color features. The higher dietary fiber content provided by the OFI microcapsules resulted in a superior texture profile in the Y-SD-OFI yogurt sample. According to the stability study conducted on samples of yogurt enriched with microcapsules of pink guava pulp in OFI or AV mucilages, these natural colorants can be recommended for the formulation of functional (antioxidant + high fiber content) Greek-type yogurts.

In summary, it was shown that the use of OFI and AV mucilage microparticles, enriched in guava lycopene, has great potential as an ingredient and natural functional colorant in the production of milk-derived products, with the ability to substitute artificial colorants for stable bioactive compounds with antioxidant properties and dietary fiber, beneficial for the health of the consumer. 

## Figures and Tables

**Table 1:** Total carotenoid content (TCC), antioxidant capacity (TEAC), and total dietary fiber content (TDFC), as well as β-carotene and lycopene quantification via HPLC–MS, for yogurt samples enriched with pulp-guava/mucilage microcapsules (Y-SD-OFI and Y-SD-AV) versus yogurt containing E110 dye (Y-C). Different superscript letters in the same row and column for each parameter indicate a statistical difference (*p* < 0.05) between samples.

Parameter	Y-SD-OFI	Y-SD-AV	Y-C
TCC ^1^	76.45 ± 1.90 ^a^	68.05 ± 2.47 ^b^	10.60 ± 0.42 ^c^
TEAC ^2^	2.65 ± 0.07 ^a^	2.20 ± 0.14 ^b^	0.40 ± 0.0 ^c^
TDFC ^3^	12.8	8.3	-
β-carotene ^4^	124	127	128
Lycopene ^4^	352	346	-

^1^ TCC is measured as µg β-carotene/g of sample in dry base. ^2^ TEAC is measured as mmol Trolox equivalents/kg of sample in dry base. ^3^ TDFC is expressed as g/100 g. ^4^ Values determined using HPLC–MS and expressed as mg/kg dry sample.

**Table 2:** Color parameters and color stability ($\Delta E^{*}$) data for yogurt samples enriched with pulp-guava/mucilage microcapsules (Y-SD-OFI and Y-SD-AV) versus yogurt containing E110 dye (Y-C). Different superscript letters in the same row and column for each parameter indicate a statistical difference (*p* < 0.05) between samples.

Color Parameter	Y-SD-OFI	Y-SD-AV	Y-C
L*	57.66 ± 0.10 ^b^	56.06 ± 0.65 ^b^	61.61 ± 0.32 ^a^
a*	2.84 ± 0.02 ^b^	3.15 ± 0.36 ^b^	13.74 ± 0.02 ^a^
b*	11.91 ± 0.07 ^b^	9.79 ± 0.56 ^b^	77.72 ± 3.04 ^a^
$C_{ab}^{*}$(chroma)	12.24 ± 0.08 ^b^	10.29 ± 0.64 ^b^	78.92 ± 2.99 ^a^
$h_{ab}$(hue)	76.58 ± 0.07 ^a^	72.22 ± 1.01 ^a^	79.96 ± 0.39 ^a^
$\Delta E^{*}$ ^1^	7.38 ± 2.32 ^c^	12.85 ± 3.58 ^b^	63.32 ± 2.64 ^a^

^1^ Color data measured after storage at 4 ± 1 °C for 25 days.

**Table 3:** Texture profile data of Y-SD-OFI, Y-SD-AV, and Y-C yogurt models.

Attribute	Y-SD-OFI	Y-SD-AV	Y-C
Firmness (g)	3.04	2.22	2.60
Consistency (g.s)	71.5	48.1	60.7
Cohesiveness (g)	−2.49	−1.67	−2.01

## Data Availability

The data presented in this study are available on request from the corresponding author.

## References

[1] FDA (2020). Overview of Food Ingredients, Additives & Colors. https://www.fda.gov/food/food-ingredients-packaging/overview-food-ingredients-additives-colors.

[2] Amchova P., Kotolova H., Ruda-Kucerova J. (2015). Health safety issues of synthetic food colorants. Regul. Toxicol. Pharmacol..

[3] Rodriguez-Concepcion M., Avalos J., Bonet M.L., Boronat A., Gomez-Gomez L., Hornero-Mendez D., Limon M.C., Meléndez-Martínez A.J., Olmedilla-Alonso B., Palou A. (2018). A global perspective on carotenoids: Metabolism, biotechnology, and benefits for nutrition and health. Prog. Lipid Res..

[4] De Freitas Santos P.D., Rubio F.T.V., da Silva M.P., Pinho L.S., Favaro-Trindade C.S. (2021). Microencapsulation of carotenoid-rich materials: A review. Food Res. Int..

[5] Rodriguez-Amaya D.B. (2019). Update on natural food pigments—A mini-review on carotenoids, anthocyanins, and betalains. Food Res. Int..

[6] Bakry A.M., Abbas S., Ali B., Majeed H., Abouelwafa M.Y., Mousa A., Liang L. (2015). Microencapsulation of Oils: A Comprehensive Review of Benefits, Techniques, and Applications. Compr. Rev. Food Sci. Food Saf..

[7] Otálora M.C., Wilches-Torres A., Castaño J.A.G. (2022). Spray-Drying Microencapsulation of Pink Guava (*Psidium guajava*) Carotenoids Using Mucilage from *Opuntia ficus-indica* Cladodes and Aloe Vera Leaves as Encapsulating Materials. Polymers.

[8] Dick M., Magro L.D., Rodrigues R., Rios A.D.O., Flôres S.H. (2018). Valorization of *Opuntia monacantha* (Willd.) Haw. cladodes to obtain a mucilage with hydrocolloid features: Physicochemical and functional performance. Int. J. Biol. Macromol..

[9] Messina C.M., Arena R., Morghese M., Santulli A., Liguori G., Inglese P. (2020). Seasonal characterization of nutritional and antioxidant properties of *Opuntia ficus-indica* [(L.) Mill.] mucilage. Food Hydrocoll..

[10] Samborska K., Boostani S., Geranpour M., Hosseini H., Dima C., Khoshnoudi-Nia S., Rostamabadi H., Falsafi S.R., Shaddel R., Akbari-Alavijeh S. (2021). Green biopolymers from by-products as wall materials for spray drying microencapsulation of phytochemicals. Trends Food Sci. Technol..

[11] De Campo C., Assis R.Q., da Silva M.M., Costa T.M.H., Paese K., Guterres S.S., de Oliveira Rios A., Flôres S.H. (2019). Incorporation of zeaxanthin nanoparticles in yogurt: Influence on physicochemical properties, carotenoid stability and sensory analysis. Food Chem..

[12] Adinepour F., Pouramin S., Rashidinejad A., Jafari S.M. (2022). Fortification/enrichment of milk and dairy products by encapsulated bioactive ingredients. Food Res. Int..

[13] Jurić S., Jurić M., Król-Kilińska Ź., Vlahoviček-Kahlina K., Vinceković M., Dragović-Uzelac V., Donsi F. (2020). Sources, stability, encapsulation and application of natural pigments in foods. Food Rev. Int..

[14] Rutz J.K., Borges C.D., Zambiazi R.C., Crizel-Cardozo M.M., Kuck L.S., Noreña C.P.Z. (2017). Microencapsulation of palm oil by complex coacervation for application in food systems. Food Chem..

[15] Gomez-Estaca J., Comunian T.A., Montero P., Favaro-Trindade C.S. (2018). Physico-Chemical Properties, Stability, and Potential Food Applications of Shrimp Lipid Extract Encapsulated by Complex Coacervation. Food Bioprocess Technol..

[16] Mihalcea L., Turturică M., Barbu V., Ioniţă E., Pătraşcu L., Cotârleţ M., Dumitraşcu L., Aprodu I., Râpeanu G., Stănciuc N. (2018). Transglutaminase mediated microencapsulation of sea buckthorn supercritical CO_2_ extract in whey protein isolate and valorization in highly value added food products. Food Chem..

[17] Iturriaga L., Quinzio C., Corvalan M., Mishima B. (2009). Study of the stability at coalescence in mucilage emulsions. Acta Hortic..

[18] Otálora M., Wilches-Torres A., Castaño J. (2021). Extraction and Physicochemical Characterization of Dried Powder Mucilage from *Opuntia ficus-indica* Cladodes and Aloe Vera Leaves: A Comparative Study. Polymers.

[19] B-290 Mini Spray Dryer Operation Manual 093001N. https://static1.buchi.com/sites/default/files/downloads/B290_OM_en_I_0.pdf?cf595fc09d939d0eb8f2bee907c35bca8feeee47.

[20] Re R., Pellegrini N., Proteggente A., Pannala A., Yang M., Rice-Evans C. (1999). Antioxidant activity applying an improved ABTS radical cation decolorization assay. Free Radic. Biol. Med..

[21] Cunniff P. (1997). Enzymatic-gravimetric method. Official Methods of Analysis of AOAC International.

[22] Di Rienzo J.A., Casanoves F., Balzarini M.G., Gonzales L., Tablada M., Robledo C.W. InfoStat Versión 2013. Grupo InfoStat, FCA, Universidad Nacional de Córdova, Argentina. http://www.infostat.com.ar.

[23] Strickland J.M., Wisnieski L., Herdt T.H., Sordillo L.M. (2021). Serum retinol, β-carotene, and α-tocopherol as biomarkers for disease risk and milk production in periparturient dairy cows. J. Dairy Sci..

[24] Gad A.S., Ghita E.I., El-Din H.M.F., Badran S.M.A., Elmessery T.M. (2015). Evaluation yogurt fortified with vegetable and fruit juice a natural source of nutritional sciences. Int. J. Food Sci. Nutr..

[25] Patel P., Jethani H., Radha C., Vijayendra S.V.N., Mudliar S.N., Sarada R., Chauhan V.S. (2019). Development of a carotenoid enriched probiotic yogurt from fresh biomass of Spirulina and its characterization. J. Food Sci. Technol..

[26] Chen W., Wang W., Guo M., Li Y., Meng F., Liu D. (2022). Whey protein isolate-gum Acacia Maillard conjugates as emulsifiers for nutraceutical emulsions: Impact of glycation methods on physicochemical stability and in vitro bioaccessibility of β-carotene emulsions. Food Chem..

[27] Jovanović J., Petronijević R.B., Lukić M., Karan D., Parunović N., Branković-Lazić I. (2017). Verification of rapid method for estimation of added food colorant type in boiled sausages based on measurement of cross section color. IOP Conf. Ser. Earth Environ. Sci..

[28] Silva M.P., Mesquita M.d.S., Rubio F.T.V., Thomazini M., Favaro-Trindade C.S. (2021). Fortification of yoghurt drink with microcapsules loaded with *Lacticaseibacillus paracasei* BGP-1 and guaraná seed extract. Int. Dairy J..

[29] Kim S., Park J.-B., Hwang I.-K. (2002). Quality Attributes of Various Varieties of Korean Red Pepper Powders (*Capsicum annuum* L.) and Color Stability during Sunlight Exposure. J. Food Sci..

[30] Mesnier X., Gregory C., Fança-Berthon P., Boukobza F., Bily A. (2014). Heat and light colour stability of beverages coloured with a natural carotene emulsion: Effect of synthetic versus natural water soluble antioxidants. Food Res. Int..

[31] Bassijeh A., Ansari S., Hosseini S.M.H. (2020). Astaxanthin encapsulation in multilayer emulsions stabilized by complex coacervates of whey protein isolate and *Persian gum* and its use as a natural colorant in a model beverage. Food Res. Int..

[32] De Queiroz J.L.C., Medeiros I., Trajano A.C., Piuvezam G., Nunes A.C.D.F., Passos T.S., Morais A.H.D.A. (2022). Encapsulation techniques perfect the antioxidant action of carotenoids: A systematic review of how this effect is promoted. Food Chem..

[33] Chung C., Rojanasasithara T., Mutilangi W., McClements D.J. (2015). Enhanced stability of anthocyanin-based color in model beverage systems through whey protein isolate complexation. Food Res. Int..

[34] Estupiñan D., Schwartz S., Garzón G. (2011). Antioxidant Activity, Total Phenolics Content, Anthocyanin, and Color Stability of Isotonic Model Beverages Colored with Andes Berry (*Rubus glaucus* Benth) Anthocyanin Powder. J. Food Sci..

[35] Borreani J., Llorca E., Quiles A., Hernando I. (2017). Designing dairy desserts for weight management: Structure, physical properties and in vitro gastric digestion. Food Chem..

[36] Hedayati S., Niakousari M., Babajafari S., Mazloomi S.M. (2021). Ultrasound-assisted extraction of mucilaginous seed hydrocolloids: Physicochemical properties and food applications. Trends Food Sci. Technol..

[37] Dai S., Corke H., Shah N.P. (2016). Utilization of konjac glucomannan as a fat replacer in low-fat and skimmed yogurt. J. Dairy Sci..

[38] Paseephol T., Small D.M., Sherkat F. (2008). Rheology and texture of set yogurt as affected by inulin addition. J. Texture Stud..

